# Humanized c-Myc Mouse

**DOI:** 10.1371/journal.pone.0042021

**Published:** 2012-07-31

**Authors:** Frank M. Lehmann, Samantha Feicht, Florian Helm, Anna Maurberger, Camilla Ladinig, Ursula Zimber-Strobl, Ralf Kühn, Josef Mautner, Armin Gerbitz, Georg W. Bornkamm

**Affiliations:** 1 Institute of Clinical Molecular Biology and Tumor Genetics, Helmholtz Center Munich, Munich, Germany; 2 Department of Gene Vectors, Helmholtz Center Munich, Munich, Germany; 3 Department of Immunology, Charité Berlin, Berlin, Germany; 4 Department of Hematology/Oncology, University of Erlangen, Erlangen, Germany; 5 Institute of Developmental Genetics, Helmholtz Center Munich, Neuherberg, Germany; 6 Department of Pediatrics, Technical University (TU) Munich and Clinical Cooperation Group Pediatric Tumor Immunology, TU Munich and Helmholtz Center Munich, Munich, Germany; University of Cincinnati, United States of America

## Abstract

**Background:**

A given tumor is usually dependent on the oncogene that is activated in the respective tumor entity. This phenomenon called oncogene addiction provides the rationale for attempts to target oncogene products in a therapeutic manner, be it by small molecules, by small interfering RNAs (siRNA) or by antigen-specific T cells. As the proto-oncogene product is required also for the function of normal cells, this raises the question whether there is a therapeutic window between the adverse effects of specific inhibitors or T cells to normal tissue that may limit their application, and their beneficial tumor-specific therapeutic action. To address this crucial question, suitable mouse strains need to be developed, that enable expression of the human proto-oncogene not only in tumor but also in normal cells. The aim of this work is to provide such a mouse strain for the human proto-oncogene product c-MYC.

**Principal Findings:**

We generated C57BL/6-derived embryonic stem cells that are transgenic for a humanized *c-Myc* gene and established a mouse strain (hc-Myc) that expresses human c-MYC instead of the murine ortholog. These transgenic animals harbor the humanized *c-Myc* gene integrated into the endogenous murine *c-Myc* locus. Despite the lack of the endogenous murine *c-Myc* gene, homozygous mice show a normal phenotype indicating that human c-MYC can replace its murine ortholog.

**Conclusions:**

The newly established hc-Myc mouse strain provides a model system to study in detail the adverse effects of therapies that target the human c-MYC protein. To mimic the clinical situation, hc-Myc mice may be cross-bred to mice that develop tumors due to overexpression of human c-MYC. With these double transgenic mice it will be possible to study simultaneously the therapeutic efficiency and adverse side effects of MYC-specific therapies in the same mouse.

## Introduction

MYC-proteins (c-MYC, MYCN and L-MYC) represent a family of transcription factors which are of particular therapeutic interest because of their strong over-expression in many human cancers [1]. The *c-MYC* gene codes for two proteins c-MYC1 (p67) and c-MYC2 (p64), that arise from alternative start codons [2]. c-MYC1 is expressed from a CUG start codon in exon 1 and c-MYC2 from the AUG codon in exon 2. In normal cells c-MYC2 is expressed in nearly all proliferating cells, whereas c-MYC1 is expressed when the cells are starved of methionine at high cell density [2]–[4]. Both c-MYC isoforms act as transcriptional activators and also as repressors of several genes. Among the c-MYC target genes are genes which regulate the synthesis of nucleotides, the cell cycle, cell proliferation, cell growth, apoptosis and ribosome biogenesis [5], [6]. c-MYC heterodimerizes with a partner protein called MAX to exert transcriptional regulation [6]. MYC-MAX dimers are able to bind to specific DNA-sequences, called E-boxes, for transcriptional regulation of genes. Negative regulators of MYC are MIZ-1 [7] and proteins of the MXD family, e.g. MAD1, MAD3, MAD4 and MXI-1 [6]–[8], which can also interact with MAX instead of MYC and block binding to E-boxes.

In several neoplasms c-MYC2 is overexpressed due to chromosomal translocation, gene amplification or abnormal transcriptional or translational regulation [1], [9]–[12]. Interestingly, by using conditional c-MYC-expressing mouse strains [13]–[16] or by inhibiting the function of c-MYC *in vivo* [17] it could be shown that c-MYC overexpression is also important for the maintenance of the cancerous stage of MYC-driven tumors. This MYC-dependency emphasizes the potential of MYC-specific cancer therapies.

Several strategies using different kinds of antisense oligonucleotides or peptide nucleic acids have been tested in cell systems and also in mouse strains for their effectiveness in inhibiting the MYC-induced proliferation of cancer cell lines [18]–[22]. In other studies small molecules were analyzed that interfere with the interaction of MYC and MAX or block the action of MYC in a different fashion [22], [23].

**Figure 1:**
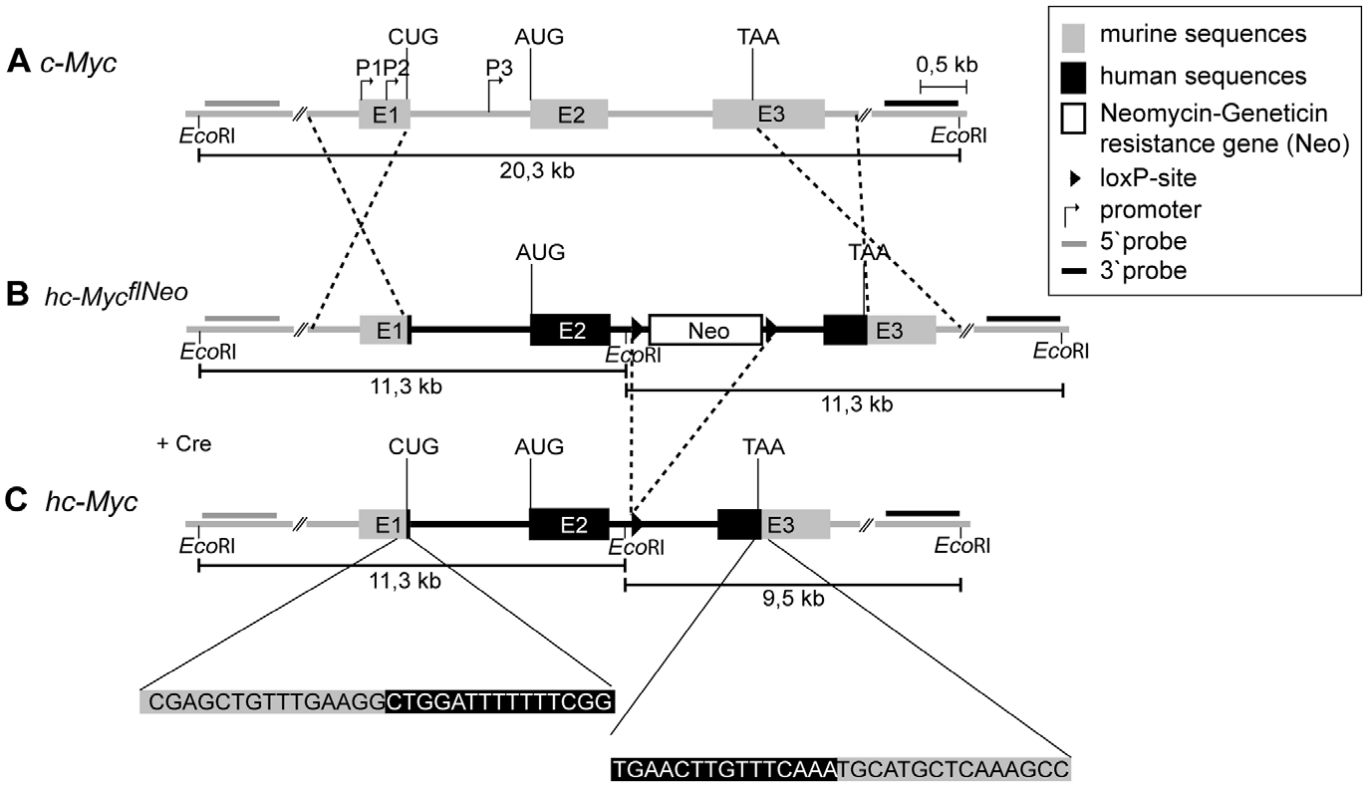
Targeting strategy for homologous recombination in ES cells. The endogenous murine *c-Myc* gene was replaced with a humanized *c-Myc* gene (*hc-Myc*). *hc-Myc* has human DNA sequences from the first translation start side (CUG) up to 69 bp downstream of the stop codon (TAA). Non-coding sequences in exon 1 and exon 3 are of murine origin so that human c-MYC is expressed under the control of murine regulatory elements. (A) Endogenous murine *c-Myc* with its three exons (E1, E2, E3) and the three promoters (P1, P2, P3). Indicated are the two start codons (CUG, AUG) and the stop codon (TAA), as well as the flanking *EcoR*I restriction sites. (B) Murine *c-Myc* locus after homologous recombination with the targeting vector. Indicated in black are human sequences, in grey murine sequences and with triangles the two loxP-sites that flank the Neomycin-Geneticin resistance gene. (C) Recombined murine *c-Myc* locus after Cre-mediated deletion of the Neomycin-Geneticin resistance gene. A piece of mouse chromosome 15 (position 61.985.920 to 61.989.995) is replaced by a piece of human chromosome 8 (position 128.748.840 to 128.753.273). Dotted lines indicate recombination events. This schematic view displays *EcoR*I restriction sites, length of fragments after *Eco*RI-digestion, the sequence in the junction region between human and murine elements and the regions where the Southern blot probes (5′ probe and 3′ probe) bind. Cre  =  Cre recombinase.

In our group we are interested in generating c-MYC-specific T cells for specific elimination of MYC-overexpressing tumor cells. One major question regarding this strategy is, whether there is a therapeutic window in which c-MYC specific T cells eliminate all tumor cells but do not cause fatal damage to healthy tissue. Likewise, for other MYC-targeting strategies it should also be of major concern to analyze in detail the adverse effects on healthy MYC-expressing tissue. However, pre-clinical *in vivo* model systems are not available to address these issues.

To fill this gap, we report here the generation of a C57BL/6 (B6) mouse strain named hc-Myc, in which the endogenous *c-Myc* gene is replaced by a humanized *c-Myc* (*hc-Myc*) gene. These animals express human c-MYC instead of the endogenous murine protein under physiological conditions. In this mouse strain strategies for targeting the human c-MYC protein can be studied. By cross-breeding humanized c-Myc mice to mouse strains that develop tumors as a consequence of overexpression of human c-MYC in pre B cells, prostate, astroglial, and B cells, like the EnMYC [24], Hi-Myc [25], GFAP/c-Myc [26] or λ-MYC mice [27] and targeting the human c-MYC protein by small molecules, siRNAs or T cells, it will become feasible to dissect therapeutic effects from adverse side effects.

## Materials and Methods

### Generation of the Targeting Vector

The B6 murine *c-Myc* locus was derived from a bacterial artificial chromosome. The murine *c-Myc* locus was retrieved as a fragment of about 14 kb by homologous recombination into plasmid pL255 (kindly provided by Ingo Burtscher), which contains a HSV-TK gene. Fragments encompassing about 500 bp each upstream and downstream of the coding region were amplified by PCR and inserted into a plasmid containing the human c*-MYC* gene, originally derived from the normal allele of a Burkitt lymphoma patient. A Neomycin-Geneticin resistance gene, flanked by loxP-sites, was inserted into the *Bgl*II restriction site in the second intron of human *c-MYC*. The human c-MYC coding region, intron 1, intron 2 and the Neomycin-Geneticin resistance cassette were inserted into the retrieval vector harboring the murine *c-Myc* locus by homologous recombination in *E. coli* using kanamycin selection. The coding sequence of the chimeric murine-human construct was thus of human origin from the CUG codon in exon 1 onwards, whereas non-coding regulatory regions in exon 1 and exon 3 were kept murine. In exon 3 the human sequence exceeds the stop codon by 69 bp. The introns 1 and 2 are of human origin. The HSV-TK gene which resided outwards the arms for homologous recombination was used for selection of embryonic stem (ES) cell clones in which homologous recombination had taken place.

### Generation of Transgenic Mice

For generation of transgenic mice B6-derived embryonic stem cells (Bruce4) were used [28]. *hc-Myc* transgenic ES cell clones were generated by standard methods and injected into Balb/c-derived blastocysts [29]. Chimeric mice were cross-bred to B6 mice. To delete the Neomycin-Geneticin resistance gene, mice were cross-bred to Cre-transgenic B6 *deleter* mice [30]. Breeding and maintaining mice was carried out under specific pathogen-free conditions (SPF).

**Figure 2:**
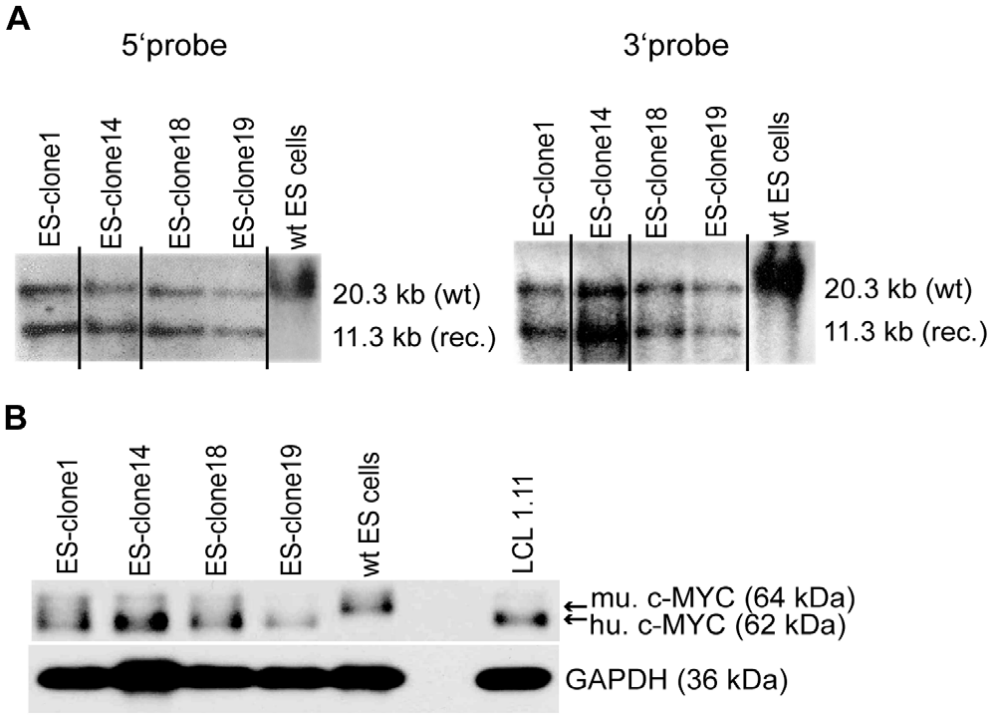
Confirmation of homologous recombination and c-MYC2 expression in ES cell clones. (A) Genomic DNA of ES cell clones 1, 14, 18 and 19 and of wildtype ES cells (wt Bruce 4) was digested with *Eco*RI. Digested DNA was analyzed by Southern blotting with a 5′ probe and a 3′ probe. (wt) DNA fragment of the wildtype *c-Myc* locus; (rec.) DNA-fragment of recombined *hc-Myc* locus. (B) Protein extracts were prepared of ES cell clones 1, 14, 18 and 19 as well as of wildtype ES cells (wt Bruce 4) and of a human lymphoblastoid cell line (LCL 1.11). Human c-MYC2 (hu. c-MYC, ca. 62 kDa) was detected with antibody clone Y69. In wildtype ES cells murine c-MYC2 (mu. c-MYC, ca. 64 kDa) was detected. For loading control an antibody specific for glyceraldehyde-3-phosphat-dehydrogenase (GAPDH; ca. 36 kDa) was used. Western blot results were reproduced five times.

### Southern Blot

For Southern blot analysis genomic DNA of ES cells was prepared by incubating ES cells o/n at 56°C in lysis buffer (10 mM Tris pH 7,4, 10 mM EDTA, 10 mM NaCl, 0,5% (w/v) SDS, 10 mg/mL Proteinase K). After cell lysis genomic DNA was precipitated with ethanol and digested o/n with *Eco*RI. DNA-fragments were separated by length on an agarose gel (0.8% agarose) and blotted o/n onto a nylon membrane. DNA was cross-linked to the membrane by baking at 80°C for 1 hour.

With radioactively labeled DNA probes that bind 5′ and 3′ site of the murine *c-Myc* locus (Figure 1) DNA-fragments containing the *c-Myc* locus were detected. Probes were radioactively labeled with 50 µCi α32-dCTP (Hartmann Analytic) by using the Random Prime Labeling Kit (GE Healthcare) according to manufacturer’s instructions.

### PCR-analysis

Mice were screened for the presence of the transgene by PCR analysis using primers specific for human and murine sequences in intron 2: humyc fwd, 5′-CACCAGGCTTAGATGTGGC-3′; mumyc fwd, 5′-GCAGCTATCCCTCACGGGA-3′ and a primer specific for a sequence in the non-translated region in exon 3: myc rev, 5′-GGCTGAAGCTTACAGTCCC-3′. For sequence analysis exon DNA was amplified by PCR with the following primers: exon1 fwd, 5′-GACTCGCCTCACTCAGCTC-3′; exon1 rev, 5′-GGCATTCGACTCATCTCAGC-3′; exon2 fwd, 5′- GTGCGTCTCCGAGATAGCA-3′; exon2 rev, 5′-GGCCCGTTAAATAAGCTGCC-3′; exon3 fwd, 5′-CACCAGGCTTAGATGTGGC-3′ and exon3 rev, 5′-GATAACCCCTTCCCATATTTG-3′.

### Western Blot

Western blot analysis of cell extracts was performed as previously described [31]. Human c-MYC was detected by using the antibody Y69, purchased from Abcam, diluted in TBST-buffer (Tris buffered saline: 0.1 M Tris/HCl pH 7.5, 0.1 M NaCl, 0.02% (v/v) tween). Equal loading was confirmed by an antibody raised against glyceraldehyde-3-phosphat-dehydrogenase (MAB374, Millipore).

**Figure 3:**
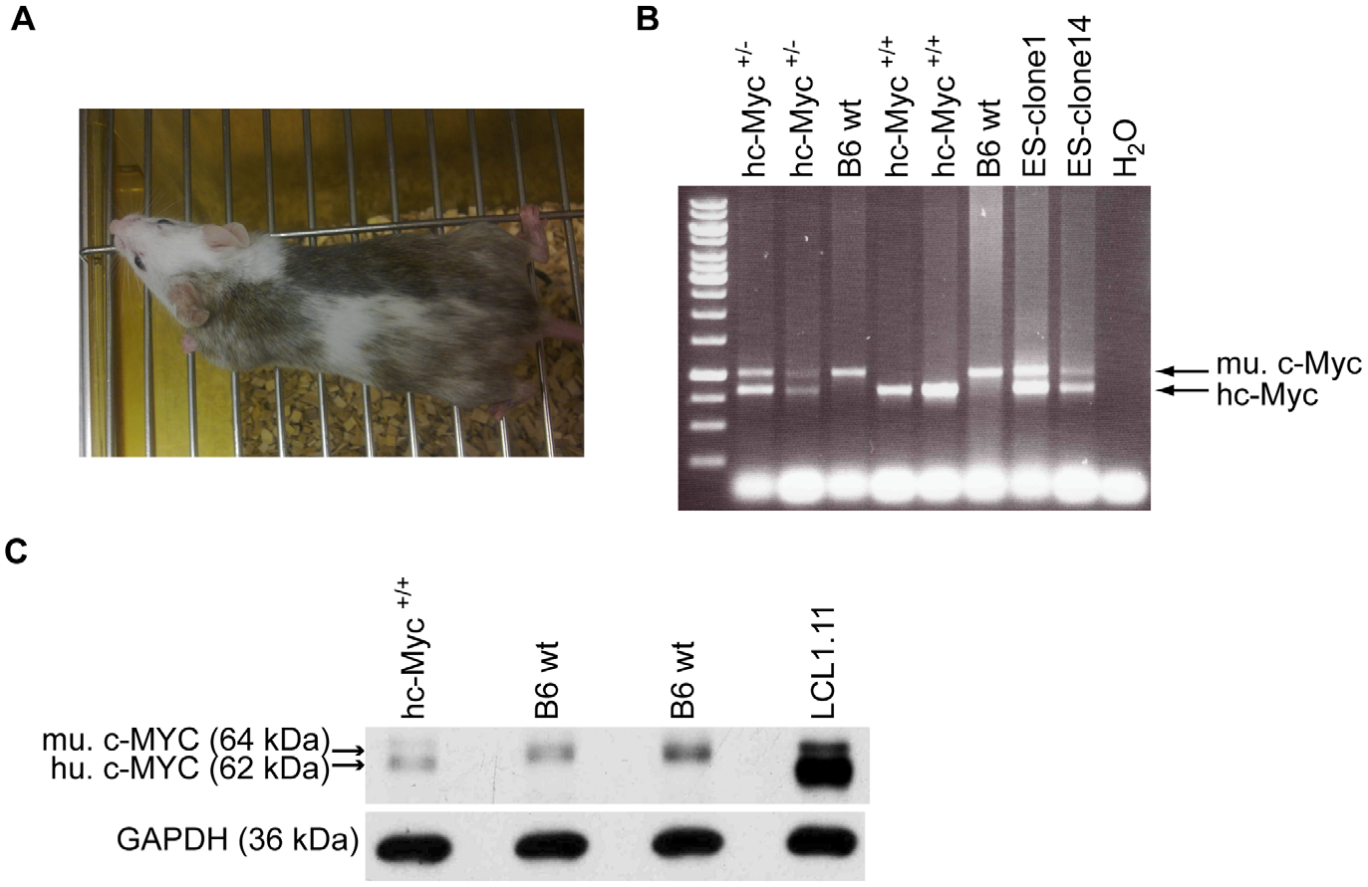
Mice transgenic for *hc-Myc*. (A) Example of a chimeric animal (chimerism of 60 to 70%) that was generated by injection of ES cell clone 14 into Balb/c blastocysts (day 3.5). (B) Chimeric animals were cross-bred to wildtype B6 mice yielding black progeny. Brother and sister matings of *hc-Myc* heterozygous mice (tested by PCR analysis) gave rise to *hc-Myc* homozygous offspring. The picture represents a PCR analysis of the genotypes of the offspring. For analysis genomic DNA was isolated from tail tissue. As controls genomic DNA of ES cell clones 1 and 14 was used. mu.c-Myc: PCR product of murine *c-Myc*. hc-Myc: PCR product of *hc-Myc*. (C) Detection of human c-MYC2 (hu. c-MYC, 62 kDa) in splenic cells of homozygous hc-Myc mice by Western blotting. In splenic cells of wildtype mice murine c-MYC2 was detected (mu. c-MYC, ca. 64 kDa). For positive control protein extracts from a human lymphoblastoid cell line (LCL 1.11) were used. Glyceraldehyde-3-phosphat-dehydrogenase (GAPDH, 36 kDa) was used as loading control.

### B Cell Isolation and *in vitro* Proliferation Assay

For isolation of mature splenic B cells, α-CD43-magnetic beads (Miltenyi Biotec) were used according to manufacturer’s instructions.

In order to analyze *in vitro* proliferation, B cells were labeled with 5 µM carboxyfluorescein succinimidyl ester (CFSE, Molecular Probes) for 5 minutes at 37°C as previously described [31]. B cells were cultivated for up to 3 days in RPMI-medium containing 10% FCS, 2 mM L-glutamine, 1 mM sodium pyruvate, 100 U/mL penicillin and 100 µg/mL streptomycin, 1 × non-essential amino acids and 50 µM ß-mercaptoethanol supplemented with the following stimuli: lipopolysaccharide (LPS; 10–25 µg/mL; *Escherichia coli* 055:B5; Sigma-Aldrich), IL-4 (10 ng/mL; mouse recombinant; Sigma-Aldrich) and α-CD40 antibody (clone HM40-3 eBioscience, 2.5 µg/mL;). Cultivation was performed in 96-well plates.

### Flow Cytometry

For flow cytometry, single cell suspensions of various lymphoid organs were prepared and analyzed with a FITC-labeled α-B220-antibody (clone RA3-6B2, BD Biosciences) and a PE-labeled α-CD3-antibody (clone 145-2C11, BD Biosciences) with the FACSCalibur™ (BD Biosciences). Data were analyzed using CELLQuest™ software (BD Bioscience). To exclude dead cells TO-PRO-3 staining was performed.

### Ethics Statement

All experiments were done complying with the German Animal Welfare act and were approved by the institutional committee on animal experimentation and the Government of Upper-Bavaria.

### Statistical Analysis

For statistical analysis a two-tailed student t-test was applied.

**Figure 4:**
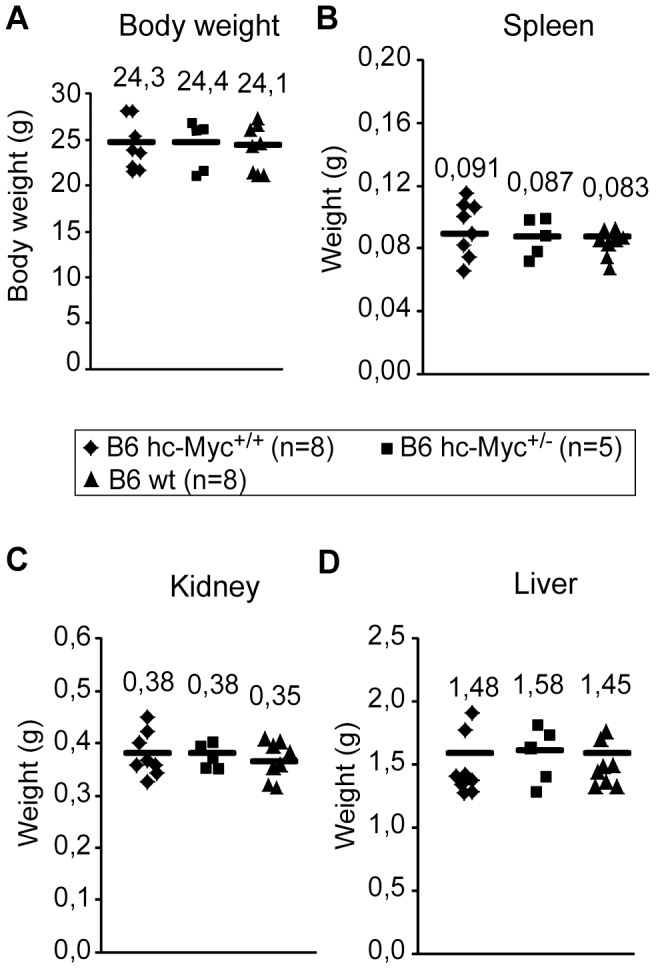
hc-Myc mice have normal body and organ weights. Body weight (A) and organ weight of spleen (B), both kidneys (C) and liver (D) were analyzed of heterozygous (B6 hc-Myc^+/−^, n = 5) and homozygous hc-Myc (B6 hc-Myc^+/+^, n = 8) mice as well as of wildtype B6 mice (B6 wt, n = 8) at the age of 10 - 11 weeks. Each symbol represents one mouse. The mean weight is indicated in the graphs with a black line and shown at the top of the symbols. Where ever small differences were observed, they were not significant.

**Figure 5:**
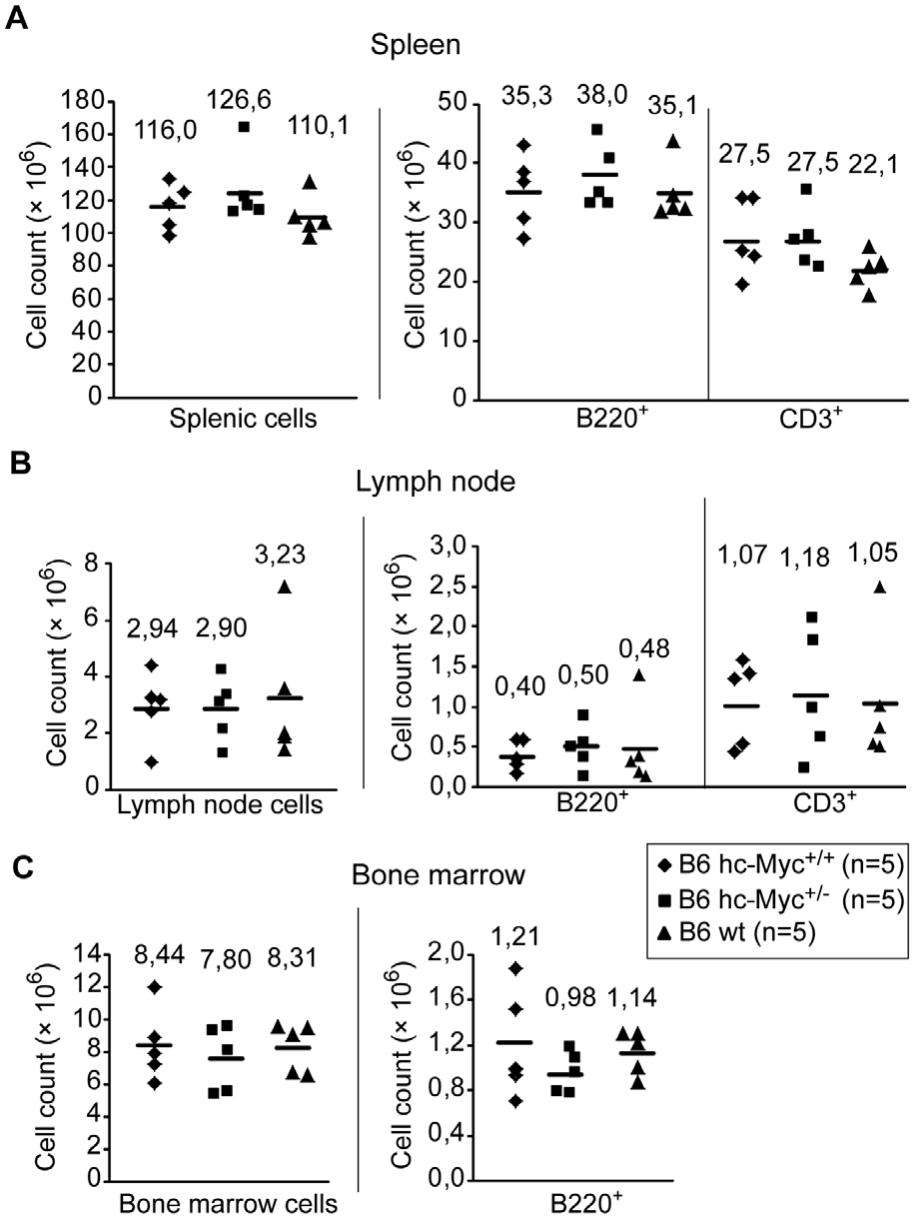
Normal cell numbers in lymphoid organs of hc-Myc mice as compared to wildtype mice. Cell numbers in the spleen (A), both inguinal lymph nodes (B) and bone marrow of one tibia (C) was analyzed of 10 – 11 week old heterozygous (B6 hc-Myc^+/−^, n = 5), homozygous (B6 hc-Myc^+/+^, n = 5) hc-Myc mice, and of wildtype B6 mice (B6 wt, n = 5). Depicted are total cell numbers of the lymphoid organs as well as B cell numbers (B220^+^) and T cell numbers (CD3^+^). Total cell numbers were counted using a Neubauer chamber. The percentage of B and T cells was determined by flow cytometric analysis. Mean values are indicated in the graph with black lines and shown at the top of the symbols. Where ever small differences were observed, they were not significant.

**Figure 6:**
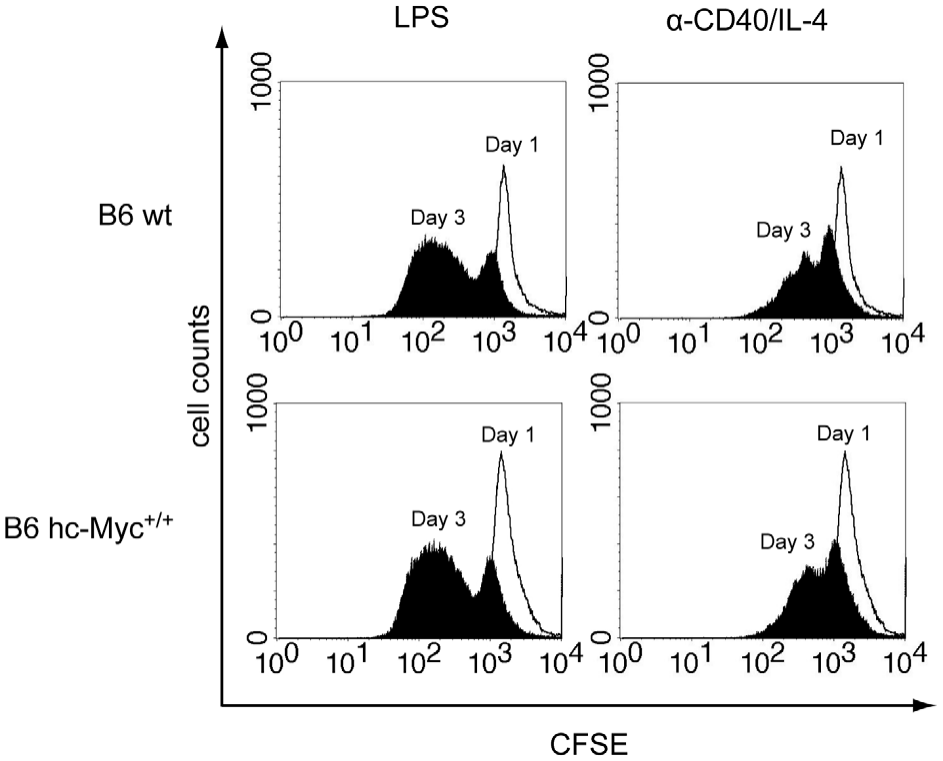
No difference in the rate of proliferation of splenic hc-Myc and wildtype B cells. Splenic B cells were isolated by magnetic cell separation from homozygous hc-Myc mice (B6 hc-Myc^+/+^) and wildtype B6 mice (B6 wt). For analyzing rate of proliferation, CFSE-stained splenic B cells were stimulated with LPS or anti-CD40 and IL-4. Proliferation was analyzed on day 1 and day 3 by flow cytometric analysis. Depicted are analyzes of living cells (TO-PRO-3 negative) on day 1 and day 3.

## Results

### Targeting Strategy

In order to generate a mouse strain in which the human c-MYC protein is expressed under physiological regulation, we integrated a humanized *c-Myc* (*hc-Myc*) gene by homologous recombination into the endogenous murine *c-Myc* locus in embryonic stem (ES) cells. The *hc-Myc* gene is composed of human c-MYC-coding sequences and human intron sequences, whereas the non-coding, regulatory regions are of murine origin (see Figure 1). This is expected to lead to normal regulation of the transgenic *c-Myc* gene by mouse regulatory elements. The human sequence encompasses the region from the CUG start codon in exon 1 up to 69 bp downstream of the stop codon so that both isoforms of the human c-MYC protein can be expressed, c-MYC1 starting from the alternate start codon CUG in exon 1 and c-MYC2 starting from AUG in exon 2. The transgene was flanked by arms for homologous recombination that enabled specific integration into one allele of the endogenous murine *c-Myc* locus in ES cells.

A Neomycin-Geneticin resistance gene was cloned into the second intron of the transgene for positive selection. The resistance gene was flanked by loxP-sites and could be deleted by cross-breeding the mice to *deleter* mice that express the Cre-recombinase ubiquitously from early embryonic stages on.

### Generation of Recombinant B6 ES Cells, Expressing Human c-MYC

For generation of transgenic hc-Myc mice, ES cells were transfected with the linearized *hc-Myc* transgene and ES cell clones in which the *hc-Myc* gene was integrated into one *c-Myc* allele by homologous recombination were selected by using Geneticin and Ganciclovir. For verification of homologous recombination a Southern blot analysis was performed. Figure 1 shows that the endogenous murine *c-Myc* locus is flanked by two *Eco*RI-restriction sites. Since the recombined *c-Myc* locus carries an additional internal *Eco*RI-restriction site, it is possible to distinguish between the endogenous and the recombined *c-Myc*-locus by *Eco*RI-digestion of genomic DNA and Southern blotting, as indicated in Figure 1. For the endogenous locus one large band (ca. 20 kb) should be detected in the Southern blot, whereas the same digestion of the recombined locus results in two smaller fragments, a 5′ fragment and a 3′ fragment (each ca. 11 kb).

Figure 2A shows an example of a Southern blot analysis of four selected ES cell clones. As a negative control wildtype ES cells were used. All four clones showed a band for the wildtype *c-Myc* allele and a second band for the 5′- (5′ probe) as well as the 3′- (3′ probe) fragment of the recombined locus. Overall 27 ES cell clones could be identified, that had shown this pattern of bands in the Southern blot and thus carried the *hc-Myc* transgene integrated into one wildtype *c-Myc* allele.

Additionally we analyzed by Western blotting whether these four ES cell clones expressed the human c-MYC protein. As positive control the EBV-immortalized human lymphoblastoid cell line LCL1.11 was used. As shown in Figure 2B, all four clones expressed the human c-MYC protein (62 kDa), whereas in wildtype ES cells only murine c-MYC of slightly higher molecular weight (64 kDa) was detectable [32]. Furthermore, to verify the human sequence of the transgene and to exclude point mutations, the coding sequence of the *hc-Myc* gene was sequenced in ES cells.

### Generation of the hc-Myc Mouse Strain

The ES cell clones 1, 14, 18 and 19 carrying the *hc-Myc* gene integrated into one allele of the endogenous *c-Myc* locus were used for injection into Balb/c blastocysts. From ES cell clones 1, 14 and 19 we could establish chimeric offspring, but only two chimeras deriving from ES cell clone 14 gave rise to transgenic offspring.

Figure 3A shows an example of one chimeric mouse derived from clone 14. This chimeric mouse was cross-bred to B6 animals. Brother-sister matings of their offspring gave rise to the mouse line with the homozygous *hc-Myc* locus. PCR analysis of tail DNA of the offspring revealed that some mice carried the *hc-Myc* transgene in a heterozygous and others in a homozygous fashion (Figure 3B). As also proven by PCR-analysis, both copies of the endogenous murine *c-Myc* gene were deleted in homozygous animals. This indicates that substitution of the murine *c-Myc* gene, whose deletion is embryonically lethal in mice, with the humanized gene is compatible with life [33].

In hc-Myc mice expression of the human c-MYC protein was additionally confirmed by Western blotting, as shown in Figure 3C. The coding sequence of the *hc-Myc* gene in transgenic mice was finally verified again by PCR amplification and sequencing of the amplification products.

### hc-Myc Mice Display a Normal Phenotype

Progeny were born in the expected mendelian ratio and were apparently normal with respect to fertility, litter size, pre- and postnatal development. In experiments with hypomorphic murine *c-Myc* alleles it has been shown previously that diminished c-MYC expression in mice resulted in reduced body weight, reduced weight of all organs and reduced proliferation of lymphoid cells *in vitro* [34]. Thus, if the human c-MYC protein is unable to fully replace its murine homologue, one should expect a similar phenotype. Therefore, heterozygous and homozygous hc-Myc mice were analyzed for these parameters.

As shown in Figure 4, the body weight and the weights of the spleen, kidney and liver was comparable between heterozygous and homozygous hc-Myc mice, as well as wildtype B6 animals. Additionally, the number of total cells, B cells (B220^+^) and T cells (CD3^+^) was determined in the spleen, inguinal lymph nodes and in the bone marrow (Figure 5). In these experiments again no difference between the three groups of mice was detected. Finally, the *in vitro* proliferation of splenic B cells was analyzed after stimulation with LPS, or α-CD40 and IL-4 (Figure 6). Apparently, B cells derived from homozygous hc-Myc mice showed similar proliferation rates as B cells derived from B6 wildtype mice after stimulation with either LPS or α-CD40 and IL-4. Taken together, our results show that the human c-MYC protein substitutes for the functions of its murine ortholog. This indicates that hc-myc mice are ideal candidates for studying the adverse effects of therapies in which the human c-MYC-protein is targeted.

## Discussion

We describe the generation of a mouse strain that contains a humanized *c-Myc* (*hc-Myc*) gene integrated into the murine *c-Myc* locus and expresses the human c-MYC protein. Thus, in the homozygous situation the endogenous murine *c-Myc* gene is deleted. Homozygous hc-Myc mice have a normal phenotype with respect to body weight, organ weights and cell numbers in lymphoid organs, and fertility indicating that the human c-Myc protein can functionally replace its murine ortholog.

The *hc-Myc* gene contains human coding sequences and murine regulatory sequences upstream of the start codon in exon 1 and downstream of the stop codon in exon 3. To redirect homologous recombination to the sequences upstream and downstream of the coding region and to prevent additional recombination events within the introns, the humanized transgene also harbors human intron sequences. As regulatory sequences for the less frequently used promoter P3 may reside in the introns of the *Myc* gene [35], promoter P3 may not be regulated in hc-Myc mice in exactly the same fashion as in the endogenous murine *c-Myc* locus. Yet, these small differences may apparently not be important for two reasons. Firstly, the differences in the regulation of the P3 promoter between the humanized and endogenous *c-Myc* gene on *Myc* transcription and translation, if they exist, are probably minimal because human and murine intron sequences are relatively similar. Secondly and most importantly, 95% of transcripts are normally generated from promoter P1 and P2 which reside upstream of the coding region in exon 1 [35]. Thus, it may be anticipated that in hc-Myc mice P1 and P2 are under the control of the regulatory elements of the endogenous murine locus.

The hc-Myc mouse strain was generated for the analysis of therapeutic prospects of c-MYC targeting strategies. To this end, it will be useful to cross-breed hc-Myc mice with animals already available that develop tumors due to an overexpression of human c-MYC in pre B, B cells, prostate cells and astroglial cells [24]–[27]. In this situation the animals shall develop tumors that overexpress human c-MYC and shall express human c-MYC also in healthy cells with high proliferative and regenerative capacity, like hematopoietic and intestinal epithelial cells. Thus it will be possible to figure out in this pre-clinical model whether a therapeutic window exists for various strategies targeting human c-MYC in malignant cells. For strategies using DNA or RNA oligonucleotides as well as for strategies using peptide nucleic acids it will be important to verify their location of binding. If they bind to coding sequences in the exon or to intron sequences of the human *c-MYC* gene they should have specificity for the *hc-Myc* transgene and should be applicable in this mouse strain. Especially for the evaluation of strategies that target the human *c-MYC* gene product by antigen-specific T cells, the hc-Myc mouse will be useful and necessary. We could show that it is possible to induce a T cell response against the human c-MYC protein in wildtype B6 mice by vaccination with c-MYC peptides covering non homologous regions between murine and human c-MYC (Helm et al., unpublished). Furthermore one immunogenic CD8^+^ T cell epitope of the human c-MYC protein could be identified in C57BL/6 mice that can be used for exploring the feasibility of c-MYC-specific T cell therapies in hc-Myc mice.
